# Contribution of rare coding mutations in *CD36* to type 2 diabetes and cardio-metabolic complications

**DOI:** 10.1038/s41598-019-53388-8

**Published:** 2019-11-20

**Authors:** David Meyre, Edward J. Andress, Tanmay Sharma, Marjolein Snippe, Hamza Asif, Arjuna Maharaj, Vincent Vatin, Stefan Gaget, Philippe Besnard, Hélène Choquet, Philippe Froguel, Kenneth J. Linton

**Affiliations:** 1 Department of Health Research Methods, Evidence, and Impact, McMaster University, Hamilton, Canada; 2 Department of Pathology and Molecular Medicine, McMaster University, Hamilton, Canada; 3 CNRS UMR8199, Pasteur Institute of Lille, Lille University, Lille, France; 4 Centre for Cell Biology and Cutaneous Research, Blizard Institute, Barts and The London School of Medicine and Dentistry, Queen Mary University of London, London, United Kingdom; 5 UMR Lipides/Nutrition/Cancer U1231 INSERM/University Bourgogne-Franche Comté/AgroSupDijon, Dijon, France; 6 Kaiser Permanente Northern California (KPNC), Division of Research, Oakland, California United States of America; 7 Department of Genomics of Common Disease, Imperial College London, London, United Kingdom

**Keywords:** Genetic association study, Diabetes complications

## Abstract

We sequenced coding regions of the cluster of differentiation 36 (*CD36*) gene in 184 French individuals of European ancestry presenting simultaneously with type 2 diabetes (T2D), arterial hypertension, dyslipidemia, and coronary heart disease. We identified rare missense mutations (p.Pro191Leu/rs143150225 and p.Ala252Val/rs147624636) in two heterozygous cases. The two *CD36* mutation carriers had no family history of T2D and no clustering of cardio-metabolic complications. While the p.Pro191Leu mutation was found in 84 heterozygous carriers from five ethnic groups from the genome aggregation database (global frequency: 0.0297%, N = 141,321), only one European carrier of the p.Ala252Val mutation was identified (global frequency: 0.00040%, N = 125,523). The Pro191 and Ala252 amino acids were not conserved (74.8% and 68.9% across 131 animal species, respectively). *In vitro* experiments showed that the two CD36 mutant proteins are expressed and trafficked to the plasma membrane where they bind modified low-density-lipoprotein (LDL) cholesterol as normal. However, molecular modelling of the recent CD36 crystal structure showed that Pro191 was located at the exit/entrance gate of the lipid binding chamber and Ala252 was in line with the chamber. Overall, our data do not support a major contribution of *CD36* rare coding mutations to T2D and its cardio-metabolic complications in the French population.

## Introduction

Type 2 diabetes (T2D) is on the rise worldwide^1^. According to the International Diabetes Federation, diabetes affected over 415 million adults globally in 2015, over 90% having T2D^2^. T2D is characterized by systemic insulin resistance and progressive beta-cell dysfunction that results in chronic high blood glucose^3^. T2D is a risk factor for several cardio-metabolic complications such as dyslipidemia, hypertension and cardiovascular disease, as well as other severe conditions like diabetic neuropathy, nephropathy, and retinopathy^4^. As such, T2D presents an increased risk of mortality and a lower quality of life for affected individuals, and an increased economic burden for societies^3,5,6^. Despite the availability of several therapeutic options, effective management of T2D and its cardio-metabolic complications has proven challenging due to its multifactorial nature^3,7^. Hence, a better understanding of the factors linking T2D to the other cardio-metabolic complications could potentially help improve prediction, prevention and management strategies^7^.

While several shared environmental factors, such as smoking, high-fat diet, and sedentary lifestyle, connect T2D with other components of the metabolic syndrome, heritability studies have evidenced a shared genetic contribution to cardio-metabolic clustering^8^. In that context, the cluster of differentiation 36 (CD36), a multifunctional class B scavenger receptor, has been identified as a promising candidate for T2D and its complications^9–12^. Given the ubiquitous expression of *CD36* across different cells and tissues and its pivotal role in glucose homeostasis and lipid metabolism, this signaling molecule links insulin resistance, obesity and T2D to dyslipidemia, atherosclerosis, and arterial thrombosis^9–14^. Genetic manipulations of *Cd36* in rodent/rat models have indicated an important role of this molecule in insulin resistance, glucose intolerance, dyslipidemia, hypertension, and coronary heart disease^11,15,16^. Increased levels of soluble CD36 (sCD36) in plasma has been strongly associated with insulin resistance, T2D, dyslipidemia, and atherosclerosis in humans^17–21^. Rare loss-of-function coding mutations in *CD36* confer impaired fatty acid metabolism, glucose intolerance, type 2 diabetes, atherosclerosis, arterial hypertension, and cardiomyopathy in humans^22^. A rare nonsense mutation (p.L360X) in *CD36* that impairs binding of CD36 to its ligand acetylated-low density lipoprotein was found in a French pedigree^23^. The mutation was associated with a non-fully penetrant autosomal dominant form of insulin resistance, T2D, hypertension and premature coronary heart disease^23^.

To gain more insight into the contribution of *CD36* rare coding mutations to T2D and its cardio-metabolic complications, we screened 184 unrelated French individuals of European ancestry presenting simultaneously with T2D, arterial hypertension, dyslipidemia and history of coronary heart disease.

## Results

### Mutations detected in French individuals presenting with both T2D and cardio-metabolic complications

We sequenced 184 non-consanguineous unrelated French individuals of European ancestry presenting simultaneously with T2D, arterial hypertension, dyslipidemia and history of coronary heart disease. Participants displayed an average age of 63.5 ± 10.0 years and an average BMI of 30.7 ± 5.8 kg/m^2^. Male participants represented 66.8% of the sample. All the genetic variants identified in the 184 probands are reported in Supplementary Table 1. We focused our attention on the rare coding mutations with a minor allele frequency (MAF) <1% as the low allele frequency of an amino acid variant can, by itself, serve as a predictor of its functional significance^24^. We identified two rare missense mutations (p.Pro191Leu/rs143150225 and p.Ala252Val/rs147624636) in two heterozygous carriers (MAFs: 0.27%, Tables 1, 2). The heterozygous carrier of the p.Pro191Leu mutation was a 60 year-old male with a BMI of 38.0 kg/m^2^. The heterozygous carrier of the p.Ala252Val mutation was a 57 year-old male with a BMI of 29.7 kg/m^2^. While we did not have access to the DNA of relatives to perform co-segregation studies, we retrieved self-reported information on the family history of diseases of the parents and siblings by the two probands. The carrier of the p.Pro191Leu mutation did not report a family history of T2D. The mother and the siblings, but not the father, had a history of hypertension. The father and the siblings, but not the mother, had a history of obesity. The carrier of the p.Ala252Val mutation did not report a family history of T2D, hypertension or obesity.

**Table 1:** List of rare coding mutations identified in the *CD36* gene.

Chromosome position (GRCh38/hg38)	SNP change	Amino-acid change	dbSNP	Gene position	Number of mutation carriers
chr7: 80663132	c > t	p.Pro191Leu	rs143150225	exon 6	1 heterozygote
chr7:80669959	c > t	p.Ala252Val	rs147624636	exon 9	1 heterozygote

**Table 2:** Frequency of rare coding mutations in the *CD36* gene in the French case, FREX control, gnomAD European global and control populations.

Variant	Number of heterozygous carriers in French cases (frequency %)	Number of heterozygous carriers in FREX controls (frequency %)	Number of heterozygous carriers in global gnomAD Europeans (frequency %)	Number of heterozygous carriers in control gnomAD Europeans (frequency %)	FREX *P*-value*	gnomAD global *P*-value*	gnomAD control *P*-value*
p.Pro191Leu	1 (0.27%)	1 (0.088%)	54 (0.035%)	18 (0.029%)	p = 1	p = 0.30	p = 0.24
p.Ala252Val	1 (0.27%)	0 (0%)	1 (0.00040%)	0 (0%)	p = 0.35	p = 0	p = 0
Total	2/184	1/566	55/77,061	18/31,413	p = 0.30	p = 0.0002	p = 0.00005

^*^Yate’s chi-square test.

### Identification of the *CD36* p.Pro191Leu and p.Ala252Val mutations in the French Exome (FREX) project

We then investigated the prevalence of the p.Pro191Leu and p.Ala252Val mutations in the FREX database. Individuals recruited in the FREX project are healthy, French adults and hence, can be used as controls to compare the relative frequency of the two identified mutations with our 184 French cases, with limited risk of bias due to population stratification^25^. One heterozygous p.Pro191Leu mutation carrier was identified among the 566 French control individuals from the FREX project (MAF: 0.088%, Table 2). In contrast, the p.Ala252Val mutation was not observed in the FREX project. A non-significant enrichment in p.Pro191Leu and p.Ala252Val mutations was observed in the French probands within our study, as compared to the control individuals from FREX (cumulative frequency: 0.54% *versus* 0.088%, p = 0.30). Similar results were observed when the two mutations were analyzed separately (p.191Leu frequency: 0.27% *versus* 0.088%, p = 1; p.252Val frequency: 0.27% *versus* 0%, p = 0.35).

### Identification of the *CD36* p.Pro191Leu and p.Ala252Val mutations in the Genome Aggregation Database (gnomAD)

We then investigated the prevalence of the p.Pro191Leu and p.Ala252Val mutations in the gnomAD database. GnomAD, to our knowledge, is the largest multiethnic, whole-genome/whole-exome sequencing resource that is publically available, and as such, it was used to estimate the prevalence of the two mutations across diverse ethnicities. Eighty four heterozygous p.Pro191Leu mutation carriers were identified from all ethnic groups (77 from exomes and 7 from genomes) including European (54/77,061; MAF: 0.035%), South Asian (4/15,307; MAF: 0.013%), East Asian (7/9,960; MAF: 0.035%), Africans/African American (8/12,481; MAF: 0.032%), Latino (9/17,720; MAF: 0.025%), other (2/3,609; MAF: 0.028), but not Ashkenazi Jewish participants (0/5,183) MAF: 0.00), with a total population of 141,321 (global MAF: 0.0297%, Table 2).

Investigation of the prevalence of the p.Pro191Leu in the control group of gnomAD (i.e. samples from individuals who were not selected as a case in a case/control study of common disease) revealed 33 heterozygous p.Pro191Leu mutation carriers in all represented ethnic groups (30 from exomes and 3 from genomes) including 18 in Europeans (18/31,413; MAF: 0.029%), 2 in South Asians (2/7,845; MAF: 0.013%), 3 in East Asians (3/4,972; MAF: 0.030%), 5 in Africans/African Americans (5/4,868; MAF: 0.051%), 5 in Latino (5/8,679; MAF: 0.029%), 0 in Ashkenazi Jewish (0/1,179; MAF: 0) and 0 in other (0/1169; MAF:0), with a total control population of 60,125 (global MAF: 0.027%, Table 2).

Only one European heterozygous carrier of the p.Ala252Val mutation was present in the whole available gnomAD population (global MAF: 0.00040%, N = 125,523, Table 2). In comparison, there were no carriers identified of the p.Ala252Val mutation found in the control group of gnomAD. Further investigation indicated that the heterozygous carrier of the p.Ala252Val mutation was a neurological case.

A significant enrichment in p.Pro191Leu and p.Ala252Val mutations was observed in the French probands within our study, as compared to the European global population and European controls from gnomAD (cumulative frequency: 0.54% *versus* 0.035%, p = 0.0002; and 0.54% *versus* 0.029%, p = 0.00005, respectively). Associations were only significant for p.Ala252Val when the two mutations were analyzed separately: p.191Leu frequency (global): 0.27% *versus* 0.035%, p = 0.30; p.191Leu frequency (control): 0.27% *versus* 0.029%, p = 0.24; p.252Val frequency (global): 0.27% *versus* 0.00040%, p = 0; p.252Val frequency (control): 0.27% *versus* 0%, p = 0).

### Conservation of *CD36* Pro191 and Ala252 amino acids across species

After alignment, the amino acids Proline found at position 191 and Alanine found at position 252 in the human CD36 protein were examined against 131 other species. The Proline corresponding to the 191st position in human CD36 was found in 74.8% of the aligned sequences, whereas the Alanine corresponding to the 252nd position in human CD36 was found in 68.9% of the sequences. The amino acid conservation score according to the Livingston *et al*. method^26^ for Pro191 and Ala252 were 2, indicating an absence of conservation of the two amino acids across species.

### *In silico* predictions of *CD36* p.Pro191Leu and p.Ala252Val function

As the contribution of the mutations to T2D and cardio-metabolic complications was unclear based on the genetic data, we investigated the functional consequences of the mutations. The p.Pro191Leu mutation was predicted to be damaging by SIFT, MutPred2 and DANN, likely damaging by PolyPhen2 and REVEL, and benign/likely benign by FATHMM. The p.Ala252Val mutation was predicted to be damaging by SIFT, FATHMM and DANN, possibly damaging by PolyPhen2, and benign/likely benign by MutPred2 and REVEL (Table 3).

**Table 3:** *In silico* analyses of rare coding mutations in the *CD36* gene.

Mutations	SIFT	PolyPhen-2	FATHMM	MutPred2	DANN	REVEL
p.Pro191Leu	Deleterious (0.03)	Probably Damaging (0.965)	Tolerated (0.426)	Pathogenic (0.617)	Deleterious (0.997)	Likely Disease Causing (0.55)
p.Ala252Val	Deleterious (0.01)	Possibly Damaging (0.928)	Deleterious (0.970)	Benign (0.410)	Deleterious (0.999)	Likely Benign (0.293)

Score values for *in silico* prediction tools are reported into brackets.

### *CD36* p.Pro191Leu and p.Ala252Val mutations are expressed as full length receptors with similar abundance to the wild-type

**Figure 1:**
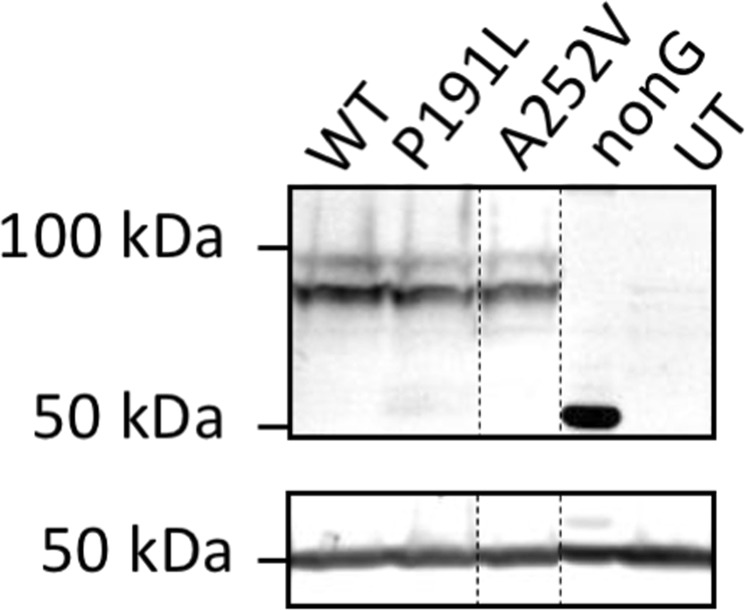
Western analysis of wild-type and variant CD36 expression. Cells were transfected with wild-type (WT) and variant CD36 constructs, as indicated. NonG is a non-glycosylatable CD36 derivative in which all ten putative glycosylation sites have been mutated. UT designates lysate from untransfected cells. CD36 expression was detected using mAb1955 (top panel). Anti-β-tubulin was used as loading control (bottom panel). Vertical dashed lines indicate the position of irrelevant lanes that were cropped from the Western blot. Full-length blots/gels are presented in Supplementary Fig. 1.

The two missense mutations identified in the coding sequence of *CD36* were engineered into the human *CD36* cDNA of pcineo-h*CD36-12his* for transient expression in HEK293T cells. Western analysis of whole cell lysates prepared from the transfected cells showed that *CD36* p.Pro191Leu and p.Ala252Val mutated cDNA sequences were transcribed, translated and glycosylated with similar efficiency as the wild-type protein (Fig. 1). CD36 migrates on SDS PAGE with an apparent molecular weight ranging between 90–110 kDa depending on the glycosylation status of the individual molecules (the *in silico* molecular weight of the protein backbone is 54 kDa, which is consistent with the migration of a non-glycosylatable derivative that is designated “nonG” in Fig. 1. In CD36-nonG all ten putative glycosylation site asparagines are mutated to glutamine (although only nine are utilized)^27^.

### Confocal microscopy shows CD36 p.Pro191Leu and p.Ala252Val mutated proteins localise to the plasma membrane

**Figure 2:**
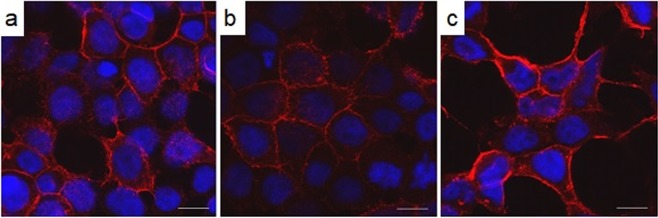
Confocal analysis of the expression patterns of wild type and variant CD36. HEK293T cells were transfected with constructs coding for wild-type human CD36 (**a**), CD36-p.Pro191Leu (**b**), and –p.Ala252Val (**c**). CD36 was immunostained using mAb1258 and an Alexa647 secondary (red); the nuclei were stained with DAPI (blue). Scalebars indicate 10 µm.

Wild-type CD36 is a heavily glycosylated, plasma membrane receptor with up to nine possible glycosylation events^27^. Core glycosylation occurs in the ER and is matured by significant modification in the terminal cisternae of the Golgi apparatus. The implication of the Western analyses is that slow migrating CD36 p.Pro191Leu and p.Ala252Val variant proteins have exited the Golgi and are either in intracellular vesicles or at the plasma membrane. To investigate further, we examined the subcellular localization of the recombinant proteins by confocal microscopy (Fig. 2). The peripheral staining in panels B and C of Fig. 2 is indicative of the presence of CD36 p.Pro191Leu and p.Ala252Val variant proteins in the plasma membrane of the cells, consistent with the expression pattern of the wild-type CD36 shown in Fig. 2a.

### Surface expression levels of p.Pro191Leu and p.Ala252Val CD36 are not different from the wild-type protein

**Figure 3:**
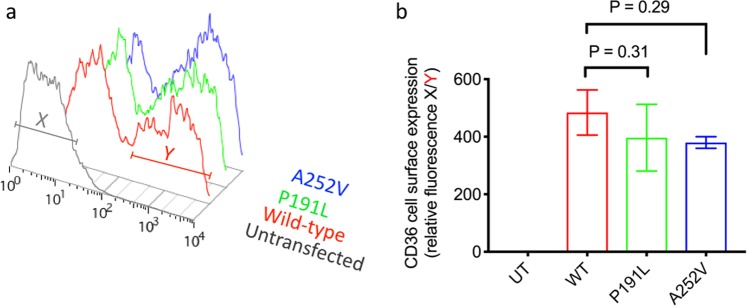
Measurement of variant CD36 expression at the cell surface by flow cytometry. Cells were transfected with wild type CD36 (WT) or variant CD36 constructs, as indicated and labelled with saturating concentrations of primary (mAb1258) and secondary (rabbit anti-mouse RPE) antibodies. R-Phycoerythrin fluorescence was detected for 10,000 cells of normal size and granularity by flow cytometry. A histogram of the red fluorescence raw data for one representative experiment is shown in (**a**). The data were quantified by gating on the population that expresses maximal CD36 for wild-type and each variant (red bar ‘Y’). The median level of fluorescence was then normalized against the median level of fluorescence of untransfected cells (grey bar ‘X’) to give a relative fluorescence intensity which can be compared across replicate data sets to produce the bar chart in (**b**). Data points represent the mean of three biological replicates and error bars describe standard error of the mean. The surface expression of CD36-p.Pro191Leu and CD36-p.Ala252Val was not statistically different to wild-type CD36 (ANOVA with Holm-Sidak’s multiple comparison test.

Using saturating conditions of primary Mab1258 antibody which recognizes an extracellular epitope on CD36, the levels of surface expression of each of the variant proteins were measured in intact live cells by flow cytometry (Fig. 3a). Statistical analysis of triplicate independent transfection experiments show that CD36 p.Pro191Leu and p.Ala252Val mutated proteins are expressed at the plasma membrane, to the same level as wild-type (Fig. 3b).

### p.Pro191Leu and p.Ala252Val CD36 bind modified-LDL with the same affinity as wild-type CD36

**Figure 4:**
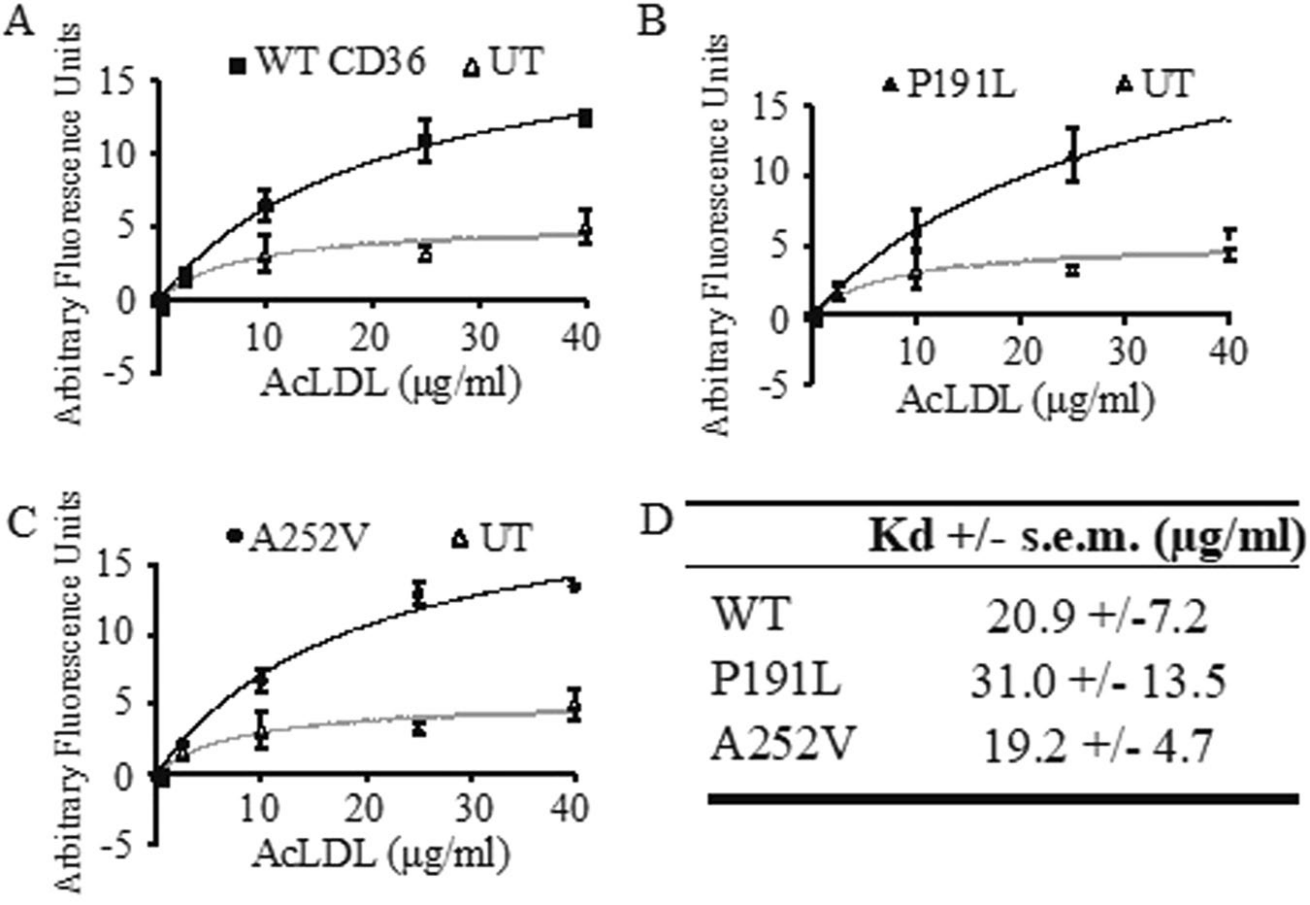
Ability of CD36 variants to bind ligand. Cells were transfected with constructs coding for CD36-wild type (**A**), CD36-p.Pro191Leu (**B**) and CD36-p.Aal252Val (**C**). Bound acLDL was measured by fluorescent-detection of the BODIPY fluorophore. Data points for each acLDL concentration were normalized internally for each transfection against 0μgml^−1^ acLDL, plotted alongside the corresponding values for an unstransfected (UT) control and best-fitted using a Langmuir adsorption isotherm equation for one-site binding. Each dataset represents the mean of three biological replicates and error bars describe standard error of the mean. Mean Kd values show no statistical difference in equilibrium binding of acLDL (**D**).

Lipoprotein binding is a property of the extracellular domain of CD36. The p.Pro191Leu and p.Ala252Val CD36 mutated protein that trafficked to the plasma membrane, and could feasibly act as a scavenger receptor, were therefore analyzed to determine if it could still bind its ligand acetylated, low-density lipoprotein (acLDL). Transiently-transfected cells, expressing either CD36 p.Pro191Leu and p.Ala252Val mutations were seeded onto 96-well plates and incubated with a range of concentrations of fluorescently-labelled acLDL (0–40μg/ml). Equilibrium binding to cells expressing wild-type CD36 was significantly above background binding to non-expressing control cells, and was best described by a binding isotherm to a single class of binding site with a mean Kd +/− SEM of 21 +/− 7 µg/ml (n = 3) (Fig. 4A). Ligand binding to CD36- p.Pro191Leu and p.Ala252Val mutated proteins was not significantly different to the wild-type (Fig. 4B,C).

### p.Pro191Leu and p.Ala252Val line the portals to the long-chain fatty acid binding cavities of CD36

**Figure 5:**
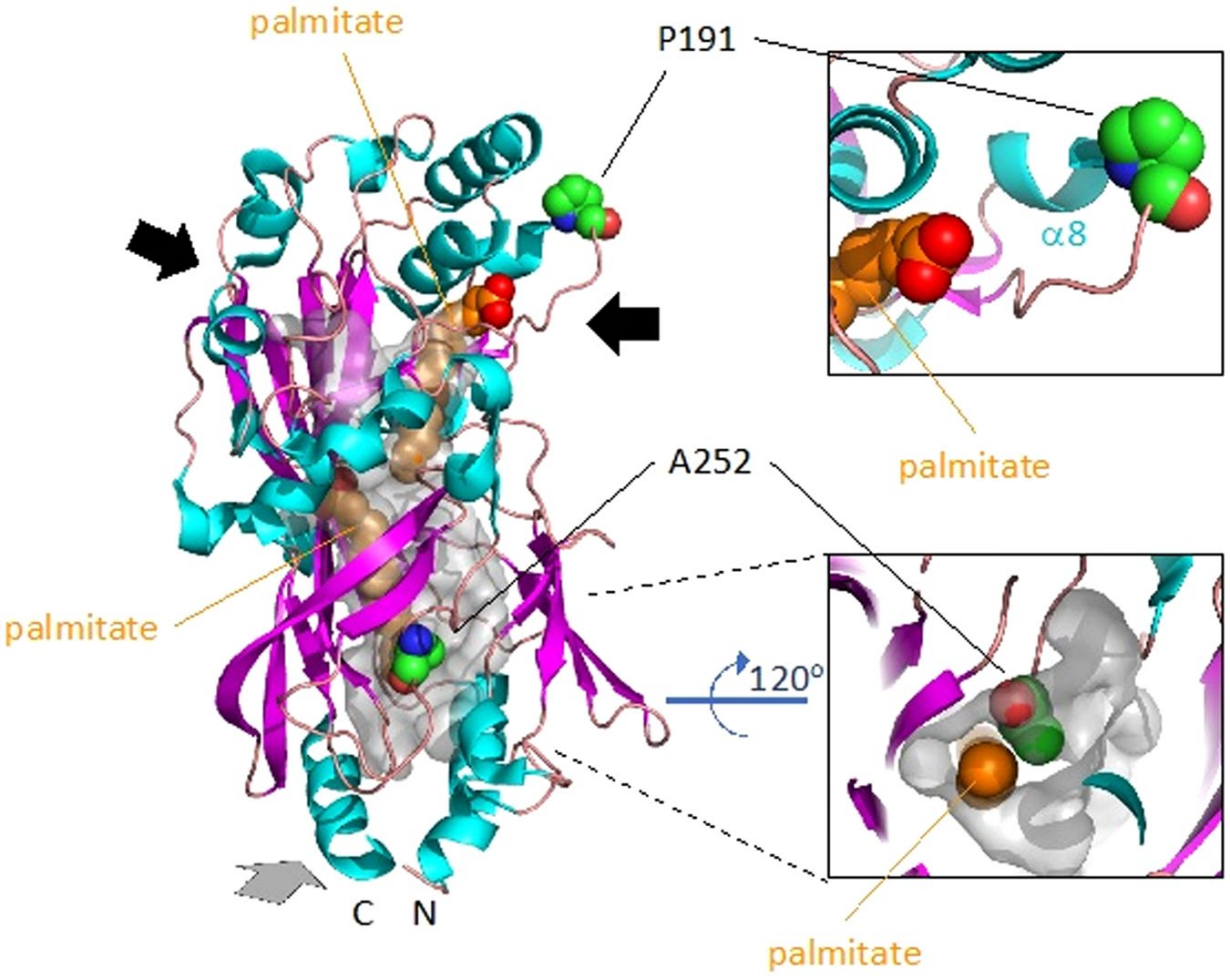
Pro191 and Ala252 are critically positioned to influence long chain fatty acid binding to CD36. The extracellular domain of human CD36 (pdb: 5lgd) is modelled in cartoon format showing the secondary structure with α-helices in cyan, β-strands in magenta and loops in salmon. The surface of the internal hydrophobic ‘Y’-shaped cavity is shown in transparent grey. The two palmitates modelled in the cavity are shown as ‘spheres’ and coloured orange (carbons) and red (oxygens). Pro191 and Ala252 are modelled in ‘spheres’ and coloured elementally with carbon in green, nitrogen in blue and oxygen in red. The three portals to the internal cavity are indicated by arrows. The grey arrow indicates a portal to the base and rear of the protein which is shown in close up from the perspective of the membrane in the bottom right panel. A close up of the upper right portal and the breaking of α-helix 8 by Pro191 is shown in the upper right panel.

Receptor-mediated endocytosis is the only mechanism that has been described for CD36 for the cellular uptake of modified-LDL^28^. Our data show that modified-LDL binding to the variant receptors is not impaired. This might suggest that the missense mutations are conservative and do not impair function, however, recent structural data proposed a different mechanism of action for long chain fatty acid uptake by the receptor which we investigated *in silico*^29^. The structure of the extracellular domain of human CD36 was resolved to 2.07 Å by X-ray crystallography^29^. The protein co-purified from HEK293 cells along with a variety of long chain fatty acids (lcFAs) identified as primarily palmitate and stearate. Two molecules of palmitate were therefore modelled into the electron densities observed within an internal ‘Y’-shaped hydrophobic cavity. The internal cavity has three portals, two distal and one proximal to the amino (N) and carboxy (C)-termini that presumably extend into the transmembrane, and which suggest that the receptor could facilitate lcFA translocation by facilitated diffusion^29^. Interrogation of this structural model (pdb: 5lgd) showed that Pro191 is located at the carboxyl-end of α-helix 8 (Fig. 5) where its role is to break this helix to allow the following loop region to form the wall of the portal leading into the cavity. Leucine, unlike proline, is not a helix breaker therefore this portal will not be properly formed and may prevent entrance or exit to the lcFA chamber. Ala252, on the other hand, lines the cavity that is proximal to membrane (Fig. 5). Substitution of the alanine by valine with its two extra methyl groups would close this cavity to prevent binding of lcFA. Both variants would therefore be very likely to impair lcFA binding or translocation through the receptor.

## Discussion

In this study, we investigated the potential contribution of *CD36* rare mutations to T2D and its cardio-metabolic complications (i.e. hypertension, dyslipidemia and history of coronary heart disease) by screening 184 French cases of European ancestry. Two rare mutations (p.Pro191Leu and p.Ala252Val, MAF: 0.27%) were found in two different participants with T2D, arterial hypertension, dyslipidemia and history of coronary heart disease. However, the two mutation carriers did not report a family history of T2D with associated cardio-metabolic complications. The p.Pro191Leu variant was found at a lower frequency in the global and control gnomAD European populations and in FREX (MAF: 0.0297%, 0.027% and 0.088%, respectively). The p.Ala252Val was found in a unique European neurological case from gnomAD and was not observed in FREX. A significant enrichment in p.Pro191Leu and p.Ala252Val mutations was observed in the French probands, when analyzed together, as compared to the global and control European individuals from gnomAD. A significant enrichment in was also observed when the p.Ala252Val mutation was analyzed separately.

The two amino acids Pro191 and Ala252 were not conserved across species. This suggests that the amino acids do not have a critical impact on fitness, but it does not exclude a functional role on CD36 function. While a majority of *in silico* software predicted damaging effects of these two mutations on CD36 function, a thorough *in vitro* functional characterization did not evidence obvious consequences on CD36 activity. More specifically, the folding and trafficking of CD36-p.Pro191Leu and CD36-p.Ala252Val are not impaired *in vitro*, their surface density is not reduced and their affinity for acetylated-LDL is not different from the wild-type protein. However, Pro191 and Ala252 are located at key positions in the crystal structure of CD36 where their substitution by leucine and valine, respectively, can possibly impair CD36 function as a facilitator of lcFA translocation. Overall, our data do not exclude a pathogenic role of the p.Pro191Leu and p.Ala252Val on CD36 function, but further investigation into the function of CD36 to understand its subunit complexity and mechanism of action, the source of the lcFA observed in the crystal structures (has it entered from the host membrane, from a neighboring cell, or from albumin or LDL in the medium) will be required to definitively answer these questions.

Very few genes have been shown to confer highly penetrant forms of T2D associated with other cardio-metabolic complications in literature so far^30^. In this context, *CD36* appeared as a promising candidate, considering previous indications of associations between *CD36* rare loss-of-function coding mutations and impaired fatty acid metabolism, glucose intolerance, type 2 diabetes, arterial hypertension, atherosclerosis, cardiomyopathy and coronary heart disease in mice and humans^11,16,22,23^. Overall, our genetic and functional experiments do not support a major contribution of *CD36* to T2D with other cardio-metabolic complications. This is in apparent contrast with a previous study by Leprete *et al*. in the French population suggesting a possible association between the p.L360X non-sense mutation in *CD36*, T2D and additional cardio-metabolic features^23^. However, it is important to note that the study by Leprete *et al*. presented some limitations including an imperfect co-segregation of the mutation with T2D and cardio-metabolic complications, as well as the use of a single pedigree^23^.

Our study has several strengths. Our selection of cases included many clinical features associated with *CD36* mutations in literature allowing for a truly representative sample. The four diseases noted in the probands (i.e. T2D, arterial hypertension, dyslipidemia and history of coronary heart disease) were diagnosed by health professionals using gold standard diagnostic criteria. We were able to perform a thorough functional characterization of *CD36* mutations including interspecies amino acid conservation analysis, *in silico* characterization, *in vitro* testing and analysis of the crystal structure. Limitations of our study include a modest sample size of cases (N = 184) which is insufficiently powered to exclude a subtle contribution of *CD36* mutations to T2D and its cardio-metabolic complications. However, this study cohort, to the best of our knowledge, is the larger samples of probands with this particular phenotypic profile (T2D, arterial hypertension, dyslipidemia and history of coronary heart disease) that has been screened for mutations in a candidate gene so far. Another limitation is that our case screening was focused on one ethnic group (French Europeans), which precludes the generalizability of our conclusions to other populations. We did not have access to the DNA of family members and therefore cannot confirm if the mutations identified in the two probands were *de novo* or transmitted by the parents. In addition, the family history of T2D, hypertension and obesity was self-reported, so this information needs to be interpreted with caution.

In conclusion, our data do not support a major contribution of rare coding mutations in *CD36* to T2D and additional cardio-metabolic complications in French individuals of European ancestry. More studies in European and other populations are needed to precise the role of *CD36* in cardio-metabolic diseases.

## Subjects and Methods

### Subjects and phenotypes

Based on previously reported observations that rare loss-of-function coding mutations in *CD36* are associated with impaired fatty acid metabolism, glucose intolerance, type 2 diabetes, atherosclerosis, arterial hypertension, and cardiomyopathy in humans^22^, we sequenced *CD36* exons in 184 French individuals of European ancestry presenting simultaneously with T2D (assessed through a fasting and/or oral glucose-tolerance test according to the 1999 World Health Organization criteria), arterial hypertension (defined as resting diastolic blood pressure ≥80 mmHg and/or systolic blood pressure ≥120 mmHg), dyslipidemia (assessed by triglyceride level ≥2.25 mmol/L and/or high-density lipoprotein cholesterol <1.03 mmol/L) and history of coronary heart disease (defined as presence of myocardial infarction, angina pectoris, bypass or the presence of a pathological Q wave on a current electrocardiogram). Our assumption was that these ‘super’ cases, with severe cardiometabolic disease profiles, may be significantly enriched in rare loss-of-function coding mutations in *CD36*^25^. We identified the 184 probands in a database of 2,526 French patients with T2D^31^. Glucose, triglycerides, and high-density lipoprotein cholesterol were measured enzymatically (Roche, Boehringer, Meylan, France). Height (m) and weight (kg) were measured in clinical centers. Standing height was measured to the nearest 0.1 cm with the participant looking straight ahead in bare feet and with his/her back against a wall. Weight was measured to the nearest 0.1 kg in light clothing. Body mass index (BMI) was calculated as weight in kilograms (kg) divided by height in meters (m) squared. Patients were recruited at the CNRS UMR8199 unit in Lille (France) and at the Endocrinology-Diabetology Department of the Corbeil-Essonnes Hospital (France).

The French Exome (FREX) project (https://www.france-genomique.org/) is a publicly available reference panel of exomes from French regions and consists in 566 healthy adult subjects recruited from six different cities.

The Genome Aggregation Database (gnomAD, http://gnomad.broadinstitute.org/) publicly available database was investigated for the presence of the two *CD36* rare coding mutations identified in the screening. GnomAD includes 125,748 and 15,708 unrelated individuals exome- and genome-sequenced, respectively, as part of various disease-specific and population genetic studies^32^. Individuals affected by severe pediatric diseases have been removed. Participants in GnomAD have been grouped in seven ethnic groups using a principal component analysis to distinguish the major axes of geographic ancestry: Europeans (N = 76,266), South Asians (N = 15,391), East Asians (N = 9,435), Africans/African Americans (N = 12,020), Latino (N = 17,210), Ashkenazi Jewish (N = 5,076) and other (N = 3,234). We investigated the prevalence of the *CD36* p.Pro191Leu in the exome-sequence and genome dataset (N = 141,321) and for p.Ala252Val mutations in 125,748 individuals with exome-sequence data only, as the data were not available for the p.Ala252Val mutation in the 15,708 genome sequences from gnomAD.

The study protocol for the patients recruited at the CNRS UMR8199 unit in Lille and the Endocrinology-Diabetology Department of the Corbeil-Essonnes Hospital has been approved by the ethical committees of Hotel-Dieu in Corbeil-Essonnes and CHRU of Lille and was performed in accordance with relevant guidelines/regulations^33^. An informed consent has been obtained from each subject before participating in the study, in accordance with the Declaration of Helsinki principles.

### Sequencing

We performed direct Sanger sequencing to screen the coding sequence of the *CD36* gene in 184 French individuals of European ancestry. The protocol was carried out using the automated ABI Prism 3730xl DNA sequencer in combination with the Big Dye Terminator Cycle Sequencing Ready Reaction kit 3.1 (Applied Biosystems). PCR conditions and primers sequences are available on request. Samples from the FREX project have been sequenced using the Agilent V5 + UTR exome capture kit and genotyped on Illumina Core Exome SNP-chip. Full details of data processing, variant calling, filtering process and variant annotation in ExAC have been previously described^34^. GnomAD was quality controlled and analyzed using the Hail open source framework (https://github.com/hail-is/hail). This data set can be accessed via the gnomAD Browser (http://gnomad.broadinstitute.org/).

### Conservation of amino acids across species

CD36 protein sequences were obtained from the UniProtKb database (https://www.uniprot.org/help/uniprotkb). The Basic Local Alignment Search Tool (BLAST) was used to search for protein sequences with the human CD36 amino acid sequence (472 amino acids) as the input^35^. Of the 250 hits found in the database, removal of redundant entries left 131 species. The sequences were imported in JalView for visualization^36^. MUSCLE alignment was used with default settings for multiple sequence alignment with the human CD36 protein sequence set as the reference^37^. Conservation scores for each column were calculated based on a method described by Livingstone *et al*.^26^. Conservation is measured as a numerical index reflecting the conservation of physico-chemical properties in the alignment. This score is measured as a numerical value ranging from 1 to 11. A score of 11 indicates no amino acid substitutions and perfect conservation amongst the 131 species. The next highest score of 10 indicates those columns with amino acid variation that preserves the physico-chemical properties^26^. Scores below 10 indicate the absence of conservation of the amino acid across species.

### In silico *analysis*

The p.Pro191Leu and p.Ala252Val mutations are listed as rs143150225 and rs147624636 in the National Center for Biotechnology Information database (NCBI) dbSNP 147. The CD36 amino acid sequence was obtained from the UniProtKB database in FASTA format. The functional consequences of the *CD36* p.Pro191Leu and p.Ala252Val mutations was predicted using the default setting of the *in silico* analysis tools SIFT^38^, PolyPhen2^39^, FATHMM^40^, MutPred2^41^, DANN^42^, and REVEL^43^. *In silico* prediction tools use neural network (SIFT, MutPred2), deep neural network (DANN), naïve Bayes classifier (PolyPhen2), hidden Markov models with support vector machine classifier (FATHMM), and random forests (REVEL) machine learning methods. DANN and REVEL integrates the score information of several *in silico* prediction tools. I*n silico* prediction tools used in this study all display score values comprised between 0 and 1. The score provided by SIFT is the normalized probability that the amino acid change is tolerated. Scores nearer zero are more likely to be deleterious. The qualitative prediction is derived from this score such that substitutions with a score <0.05 are called ‘deleterious’ and all others are called ‘tolerated’. The PolyPhen-2 score represents the probability that a substitution is damaging, so values closer to 1 are more confidently predicted to be deleterious (opposite to SIFT). Scores greater than 0.908 are listed as “Probably Damaging”, greater than 0.446 and less than or equal to 0.908 are listed as, “Possibly Damaging”, less than or equal to 0.446 are listed as “Benign” conventionally. DANN and FATHMM scores are also reported from 0 to 1 with scores closer to 1 being considered more deleterious. Category-optimal decision thresholds via Predict SNP2 were used to classify DANN and FATHMM scores^44^. MutPred2 scores are reported from 0 to 1 with scores closer to 1 being considered more deleterious. Variants with predicted Scores above 0.5 are considered pathogenic. Variants with higher scores in REVEL are predicted to be more likely to be pathogenic. REVEL does not provide a descriptive prediction but scores above 0.5 are conventionally labelled as ‘likely disease causing’ and scores below 0.5 as ‘likely benign’.

### Site-directed mutagenesis of CD36

Site-directed mutagenesis was used to introduce SNPs into the human cDNA for *CD36* in pCIneo-hCD36-12His^27^ using the Quickchange II kit (Stratagene) in accordance with the manufacturer’s instructions. The entire coding sequence of each mutant was confirmed by DNA-sequencing prior to experimental use. Mutagenic primer sequences are reported in Supplementary Table 2.

### Mammalian cell culture and protein expression

HEK293T cells were grown in Dulbecco’s modified Eagle’s medium (DMEM) supplemented with 10% fetal bovine serum (Gibco) at 37 °C in 5% CO_2_. Cells were transfected transiently with wild type or variant pCIneo-hCD36-12His, as described previously^45^. Twenty four hours post-transfection the cells were treated with 2 μM butyric acid (Sigma) to stimulate transcription, and cultured for a further 24 hours before lysis or harvesting.

### Glycosidase treatment

PNGase F and Endo H glycosidases were obtained from New England Biolabs and used to deglycosylate whole cell extracts as per the manufacturer’s instructions.

### Western blotting

Whole cell lysates (50 μg of protein) were prepared from transfected HEK293T cells in 1% SDS buffered with HEPES plus 100 mM NaCl and complete protease inhibitor cocktail (Roche). The proteins were separated by SDS-PAGE and transferred to PVDF membrane (Millipore). Western blots were probed with rat anti-CD36 (Mab1955; R&D systems) and mouse anti-β-tubulin (Source Bioscience UK) primary antibodies, using 1:1,000 and 1:2,000 dilutions respectively, in blocking buffer (5% dried milk in PBS plus 0.1% Tween 20). Appropriate fluorophore-conjugated secondary antibodies were obtained from Licor and used at a dilution of 1:20,000, allowing detection of labelled proteins using the Odyssey infrared imaging system (LiCor).

### Microscopy

For immunofluorescence, cells were grown and transfected on glass coverslips before being fixed using 4% formaldehyde and permeabilised with 1% triton. The samples were blocked with 5% BSA in PBS for 1 hr, then probed with anti-CD36, Mab1258 (Chemicon International) diluted 1:100 with 1% BSA in PBS for 1 hr. The sample was then washed three times in PBS before incubation with Alexa647-conjugated goat anti-mouse secondary (Invitrogen) diluted 1:1,000 with 1% BSA in PBS. Finally, the samples were washed three times in PBS before visualisation of the labelled proteins by confocal microscopy. DAPI was used at 1:2,000 to stain nuclei. Samples were analysed using a Zeiss 510 inverted confocal laser scanning microscope using a Plan-Apochromat 63x NA 1.4 objective. The Argon 364 nm laser line was used and output intensity set to 10.2% to excite DAPI; for Alexa647, the HeNe laser (633 nm) was used at 9%. For detection, the HFT UV/488/543/633 main dichroic beam splitter was used in combination with a NFT 635 Vis secondary beam splitter and a band pass filter (BP 385–470) for DAPI or a long pass filter (LP650) for Alexa647. The pinhole was set at 1.8 airy units. Operating in multi-track mode allowed for crosstalk-free imaging of the two dyes. All samples were processed using the same settings.

### Flow cytometry

Flow cytometry was carried out essentially as described previously^27^. Briefly, for each sample, 1 × 10^5^ cells were harvested, washed in PBS, blocked in PBS plus 1% fatty-acid-free BSA (Sigma), incubated with anti-CD36 (Mab1258; Chemicon International) in saturating conditions (2 μg), washed three times in PBS plus 1% BSA, incubated with a RPE-conjugated rabbit anti-mouse secondary (DAKO) in saturating conditions (4 μg) and then washed a further three times in PBS plus 1% BSA. Analysis of CD36 cell surface expression was performed on a FACScan flow cytometer from BD Biosciences. Cells were gated for normal size and granularity. Voltage of the FL-2 channel was kept constant at 378 V for all experiments. The fluorescence peak associated with maximal CD36 expression was gated as indicated for each set of experimental replicates and the median fluorescence values of cells expressing wild-type and variant CD36 compared, following internal normalisation against untransfected cells.

### BODIPY-labelled acetylated-LDL (BODIPY-acLDL) binding assay

The ligand binding assay was carried out as described previously^27^. Briefly, cells were split 24 hours post transfection into a 96-well plate and allowed to adhere for a further 24 hours. The cells were then washed, blocked and incubated with increasing concentrations (0–40μg/ml) of BODIPY-acLDL for 2hrs at 4 °C. Finally, the cells were washed and bound ligand was measured by analysis of BODIPY-fluorescence using a Synergy HT plate reader (Bio-Tek). Binding data for each biological replicate was normalised internally versus a blank for each transfection (0 μg/ml acLDL), then averaged and compared to the untransfected control to show the level of background binding. Data points were best-fitted in Graphpad Prism v4.0 using Langmuir adsorption isotherm equation for one-site binding:

$${\rm{B}}={\rm{Bmax}}\,\ast \,{\rm{X}}/{\rm{Kd}}+{\rm{X}}$$

Where B is bound ligand (relative fluorescence units), Bmax is maximal binding (relative fluorescence units), [X] is concentration of ligand (μg/ml), Kd is the concentration of ligand (μg/ml) giving half-maximal binding and is a measure of the affinity of the receptor-ligand interaction.

### Statistical analysis

*In vitro* data was expressed as mean values. Differences between groups were compared with the use of an unpaired Student’s t-test using the Graphpad prism v7 software. Yates’ chi-square tests were used to compare the allele frequencies of the p.Pro191Leu and p.Ala252Val mutations between groups. All reported *P*-values are two-sided. *P*-values of less than 0.05 were considered significant.

## Supplementary information

Supplementary information

## Data Availability

The datasets generated during and/or analyzed during the current study are available from the corresponding author on reasonable request. Additional datasets analyzed during the current study are available in the UniProtKb (https://www.uniprot.org/), gnomAd (http://gnomad.broadinstitute.org/) and FREX (https://www.france-genomique.org/) databases.
