# Supplementary material for: Contribution of rare coding mutations in CD36 to type 2 diabetes and cardio-metabolic complications
Source: Sci Rep. 2019 Nov 20;9:17123. doi: 10.1038/s41598-019-53388-8 (PMC6868229; doi:10.1038/s41598-019-53388-8)
Supplement: Supplementary file 1 — Supplementary information [file 41598_2019_53388_MOESM1_ESM.docx]

**Contribution of rare coding mutations in *CD36* to type 2 diabetes and cardio-metabolic complications**

David Meyre^1,2,3,*^, Edward J. Andress^4^, Tanmay Sharma^1^, Marjolein Snippe^4^, Hamza Asif^1^, Arjuna Maharaj^1^, Vincent Vatin^3^, Stefan Gaget^3^, Philippe Besnard^5^, Hélène Choquet^3,6^, Philippe Froguel^3,7,*^, Kenneth J. Linton^4,*^.

^1^Department of Health Research Methods, Evidence, and Impact, McMaster University, Hamilton, Canada; ^2^Department of Pathology and Molecular Medicine, McMaster University, Hamilton, Canada; ^3^CNRS UMR8199, Pasteur Institute of Lille, Lille University, Lille, France; ^4^Centre for Cell Biology and Cutaneous Research, Blizard Institute, Barts and The London School of Medicine and Dentistry, Queen Mary University of London, London, United Kingdom; ^5^UMR Lipides/Nutrition/Cancer U1231 INSERM/University Bourgogne-Franche Comté/AgroSupDijon, Dijon, France; ^6^Kaiser Permanente Northern California (KPNC), Division of Research, Oakland, California, United States of America; ^7^Department of Genomics of Common Disease, Imperial College London, London, United Kingdom.

**Corresponding authors:** Dr. David Meyre, Department of Health Research Methods, Evidence, and Impact, McMaster University, Michael DeGroote Centre for Learning & Discovery, Room 3205, 1280 Main Street West, Hamilton, ON L8S 4K1, Canada. Email: [meyred@mcmaster.ca](mailto:meyred@mcmaster.ca). Pr Philippe Froguel Centre National de la Recherche Scientifique (CNRS) UMR 8199, Institut Pasteur de Lille, University of Lille, 1 place de Verdun 59045 Lille Cedex - France. Department of Genomics of Common Disease, Imperial College London, Kensington, London SW7 2AZ, UK. Email: p.froguel@imperial.ac.uk. Pr. Kenneth J. Linton Centre for Cell Biology and Cutaneous Research, Blizard Institute, Barts and The London School of Medicine and Dentistry, Queen Mary University of London, 4 Newark Street, Whitechapel, London, United Kingdom E1 2AT. Email: [k.j.linton@qmul.ac.uk](mailto:k.j.linton@qmul.ac.uk).

**Supplementary Table 1.** List of genetic variants identified in the *CD36* gene in 184 French individuals of European ancestry presenting simultaneously with type 2 diabetes, arterial hypertension, dyslipidemia, and coronary heart disease.

| **SNP description** | **dbSNP** | **Chromosome position NCBI36/hg18** | **Gene position** | **SNP change** | **Amino acid change** | **N** |
| --- | --- | --- | --- | --- | --- | --- |
|  |  |  |  |  |  |  |
|  | - | 80,069,536 | 5'UTR | G>A | - | 1 heterozygous carrier GA |
|  | - | 80,069,559 | 5'UTR | C>T | - | 1 heterozygous carrier CT |
|  | - | 80,069,622 | 5'UTR | C>G | - | 1 heterozygous carrier CG |
|  | rs1049654 | 80,113,391 | 5'UTR | C>A | - | 56 CC, 88 CA, 40 AA |
| P191L | rs143150225 | 80,130,399 | exon 6 | C>T | P/L | 1 heterozygous carrier CT |
| P191P | rs5956 | 80,130,400 | exon 6 | G>A | P/P | 5 heterozygous carriers GA |
| A252V | rs147624636 | 80,137,211 | exon 9 | C>T | A/V | 1 heterozygous carrier CT |
|  | - | 80,140,662 | intron13 | G>A | - | 1 heterozygous carrier GA |
|  | - | 80,143,829 | 3'UTR | C>T | - | 1 heterozygous carrier CT |
|  | - | 80,144,081 | 3'UTR | C>T | - | 1 heterozygous carrier CT |
|  | rs7755 | 80,144,207 | 3'UTR | G>A | - | 60 GG, 91GA, 33 AA |

**Supplementary Table 2.** Mutagenic Primers.

| P191L-F | CCATTTTTGAGTTTGGTTCTGTATCCTGTTACTACCACAGTTGG |
| --- | --- |
| P191L-R | CCAACTGTGGTAGTAACAGGATACAGAACCAAACTCAAAAATGG |
| A252V-F | GGTACAGATGCTGTCTCATTTCCACCTTTTGTTGAGAAAAGCC |
| A252V-R | GGCTTTTCTCAACAAAAGGTGGAAATGAGACAGCATCTGTACC |


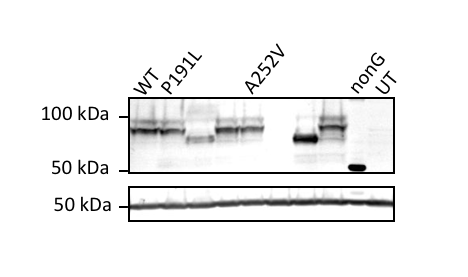


**Supplementary Figure 1.**  **Western analysis of wild-type and variant CD36 expression.** Cells were transfected with wild-type (WT) and variant CD36 constructs, as indicated. NonG is a non-glycosylatable CD36 derivative in which all ten putative glycosylation sites have been mutated. UT designates lysate from untransfected cells. CD36 expression was detected using mAb1955 (top panel). Anti-β-tubulin was used as loading control (bottom panel).
